# Crystal structure of [3-(1*H*-benzimidazol-2-yl)propano­ato-κ*N* ^3^][3-(1*H*-benzimid­azol-2-yl)propanoic acid-κ*N* ^3^]copper(I)

**DOI:** 10.1107/S2056989014026656

**Published:** 2015-01-01

**Authors:** Zhimin Liu, Shengrun Zheng, Sisi Feng

**Affiliations:** a School of Chemistry and Chemical Engineering, Shanxi University, Taiyuan 030006, People’s Republic of China; b Institute of Special Materials & School of Chemistry and Environment, South China Normal University, Guangzhou 510006, People’s Republic of China; c Institute of Molecular Science, Key Laboratory of Chemical Biology and Molecular, Engineering of the Education Ministry, Shanxi University, Taiyuan, Shanxi 030006, People’s Republic of China

**Keywords:** crystal structure, 3-(1*H*-benzimidazol-2-yl)propanoic acid, copper(I), hydrogen bonding

## Abstract

In the title compound, [Cu(C_10_H_9_N_2_O_2_)(C_10_H_10_N_2_O_2_)], the Cu^I^ ion is situated at a crystallographic centre of inversion and is coordinated in a linear environment by two benzimidazole N atoms from two symmetry-related 2-propanoic-1*H*-benzimidazole ligands. The ligands are disordered in a sense that statistically one of the carb­oxy­lic acid groups in each mol­ecule is deprotonated. In the crystal, O—H⋯O hydrogen bonds link the mol­ecules into chains along the *a-*axis direction. These chains are additionally linked into infinite two-dimensional networks in the *ab* plane by N—H⋯O hydrogen bonds.

## Related literature   

For background to benzimidazole complexes with copper(I), see: Lei *et al.* (2010 ▸). For the structures and properties of transition metal complexes with 3-(1*H*-benzimidazol-2-yl)propanoic acid ligands, see: Zheng *et al.* (2012 ▸); Zeng *et al.* (2007 ▸); Yao *et al.* (2008 ▸); Choi (2004 ▸).
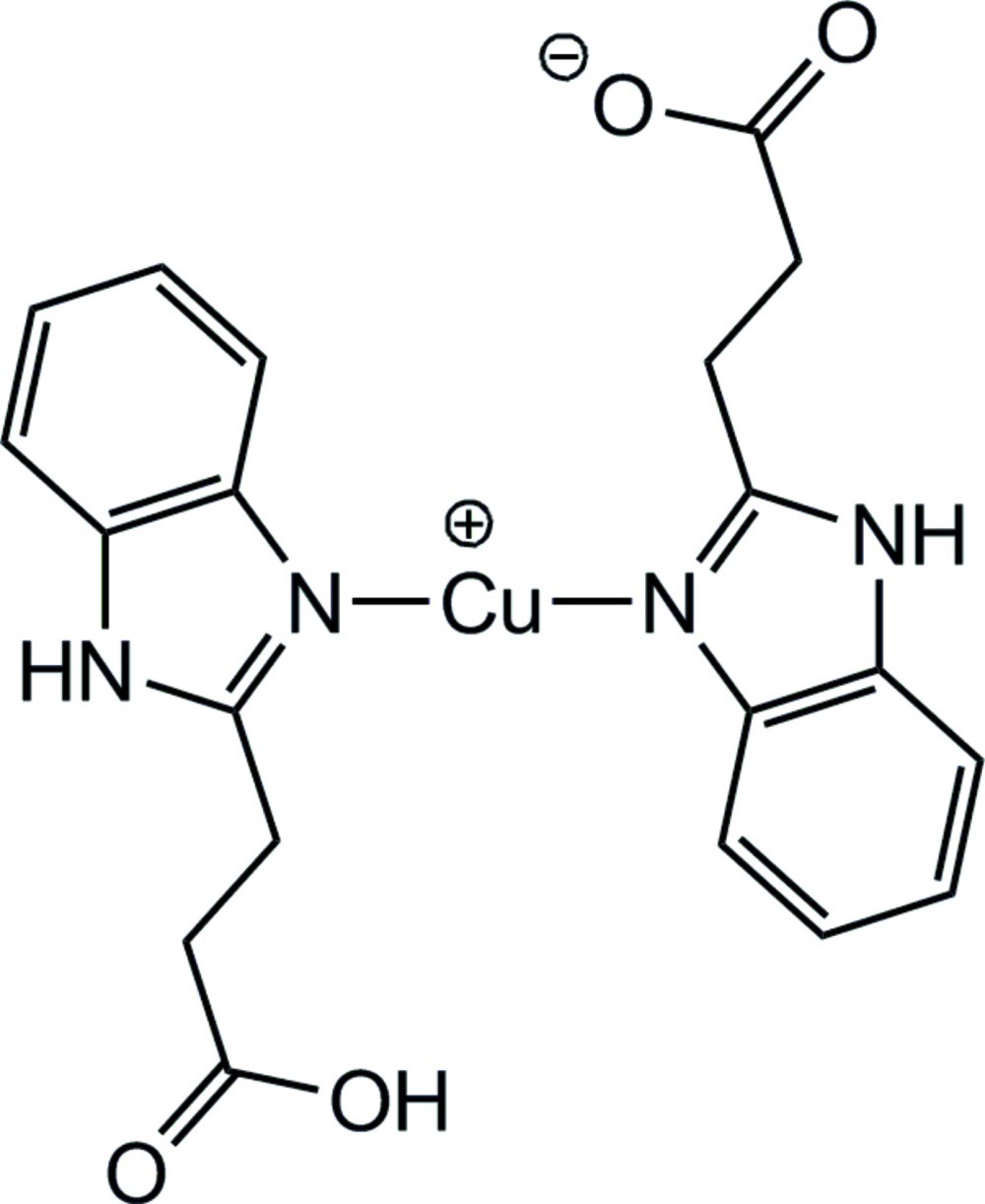


## Experimental   

### Crystal data  

[Cu(C_10_H_9_N_2_O_2_)(C_10_H_10_N_2_O_2_)]
*M* *_r_* = 442.93
Monoclinic, 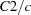

*a* = 21.137 (5) Å
*b* = 6.4979 (14) Å
*c* = 16.235 (4) Å
β = 121.949 (2)°
*V* = 1892.0 (7) Å^3^
*Z* = 4
Mo *K*α radiation
μ = 1.19 mm^−1^
*T* = 298 K
0.28 × 0.24 × 0.20 mm

### Data collection  

Bruker APEXII CCD diffractometer
Absorption correction: multi-scan (*SADABS*; Sheldrick, 1996 ▸) *T*_min_ = 0.732, *T*_max_ = 0.797
4975 measured reflections
1855 independent reflections
1370 reflections with *I* > 2σ(*I*)
*R*_int_ = 0.031

### Refinement  

*R*[*F* ^2^ > 2σ(*F* ^2^)] = 0.038
*wR*(*F* ^2^) = 0.101
*S* = 1.01
1855 reflections
134 parameters
H-atom parameters constrained
Δρ_max_ = 0.29 e Å^−3^
Δρ_min_ = −0.26 e Å^−3^

### 

Data collection: *APEX2* (Bruker, 2004 ▸); cell refinement: *SAINT* (Bruker, 2002 ▸); data reduction: *SAINT*; program(s) used to solve structure: *SHELXS97* (Sheldrick, 2008 ▸); program(s) used to refine structure: *SHELXL97* (Sheldrick, 2008 ▸); molecular graphics: *SHELXTL* (Sheldrick, 2008 ▸); software used to prepare material for publication: *SHELXTL*.

## Supplementary Material

Crystal structure: contains datablock(s) I, New_Global_Publ_Block. DOI: 10.1107/S2056989014026656/im2457sup1.cif

Structure factors: contains datablock(s) I. DOI: 10.1107/S2056989014026656/im2457Isup2.hkl

**Fig. 1.**
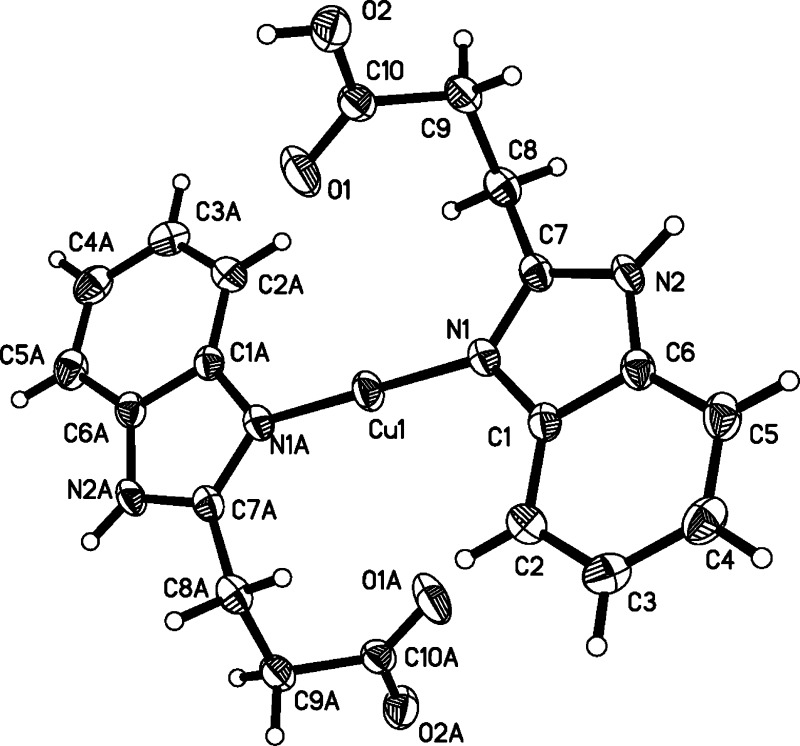
The structure of the title compound, showing 30% probability displacement ellipsoids and the atom-numbering scheme.

. DOI: 10.1107/S2056989014026656/im2457fig1.tif
The structure of the title compound, showing 30% probability displacement ellipsoids and the atom-numbering scheme.

**Fig. 2.**
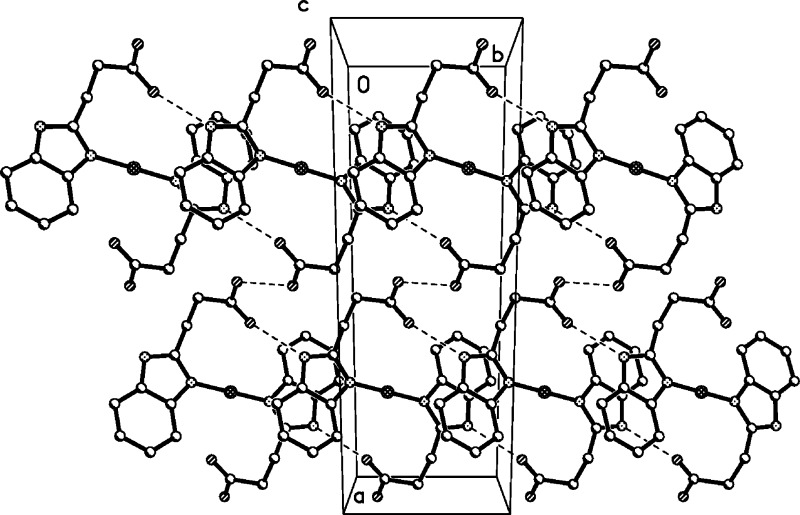
Part of the crystal structure of the title compound, showing the formation of the two-dimensional network by hydrogen bonds (dashed lines). H atoms are omitted for clarity.

. DOI: 10.1107/S2056989014026656/im2457fig2.tif
Part of the crystal structure of the title compound, showing the formation of the two-dimensional network by hydrogen bonds (dashed lines). H atoms are omitted for clarity.

CCDC reference: 1033367

Additional supporting information:  crystallographic information; 3D view; checkCIF report

## Figures and Tables

**Table 1:** Hydrogen-bond geometry (, )

*D*H*A*	*D*H	H*A*	*D* *A*	*D*H*A*
N2H2*A*O1^i^	0.86	1.97	2.725(3)	146
O2H2*B*O2^ii^	0.82	1.69	2.491(5)	166

Symmetry codes: (i) 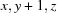; (ii) 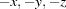.

## supplementary crystallographic information

### S1. Comment

Benzimidazole and its derivatives have been extensively used in building pharmaceutical compounds. A number of metal-benzimidazole complexes have been studied due to their potential applications (Zheng *et al.*, 2012). In this paper, we report a new structure, [Cu(C_10_H_9_N_2_O_2_)(C_10_H_10_N_2_O_2_)], which was synthesized by condensation of 3-(1*H*-benzimidazol-2-yl) propanoic acid in the presence of copper(I) chloride.

As shown in Fig1, the Cu(I) ion is situated at a crystallographic centre of symmetry and is coordinated by two N atoms from two -(2-propanoic) benzimidazole ligands in a linear environment. The ligands are disordered in a sense that statistically one of the carboxyl groups in each complex molecule is deprotonated. This means that there is one negatively charged ligand and the oxidation state of copper therefore is +1. Due to the low coordination number of Cu(I), the bond distances of Cu(I)–N (1.851 Å) is shorter than those reported recently (Lei *et al.*, 2010). In the crystal structure, O—H···O hydrogen bonds link the molecules into one-dimensional chains along the *a* axis. These chains are additionally linked into infinte two-dimensional networks in the *ab* plane by N—H···O hydrogen bonds. (Table 1 and Fig. 2).

### S2. Experimental

The ligand 3-(1*H*-benzimidazol-2-yl) propanoic acid was prepared according to a procedure described by Yao *et al.* (2008). A mixture of copper(I) chloride (0.10 g, 1.0 mmol), 3-(1*H*-benzimidazol-2-yl) propanoic acid (0.38 g, 2.0 mmol) and methanol (15 ml) was sealed in 25ml Teflon-lined stainless steel reactor and heated to 423K for 72h. Yellow block crystals of the title compound suitable for X-ray analysis were obtainted (yield: 73% based on 3-(1*H*-benzimidazol-2-yl) propanoic acid).

### S3. Refinement

H atoms bonded to C, N and O atoms were positioned geometrically and refined as riding atoms, with C–H = 0.93 (aromatic), 0.97 (CH_2_), N–H = 0.86 (NH) and O–H= 0.82 Å with U_iso_(H) = 1.2 U_eq_(C,N) and U_iso_(H) = 1.5 U_eq_(O).

### Crystal data

**Table:**

[Cu(C_10_H_9_N_2_O_2_)(C_10_H_10_N_2_O_2_)]	*F*(000) = 912
*M**_r_* = 442.93	*D*_x_ = 1.555 Mg m^−^^3^
Monoclinic, *C*2/*c*	Mo *K*α radiation, λ = 0.71073 Å
Hall symbol: -C 2yc	Cell parameters from 1118 reflections
*a* = 21.137 (5) Å	θ = 2.3–22.1°
*b* = 6.4979 (14) Å	µ = 1.19 mm^−^^1^
*c* = 16.235 (4) Å	*T* = 298 K
β = 121.949 (2)°	Block, yellow
*V* = 1892.0 (7) Å^3^	0.28 × 0.24 × 0.20 mm
*Z* = 4	

### Data collection

**Table:**

Bruker APEXII CCD diffractometer	1855 independent reflections
Radiation source: fine-focus sealed tube	1370 reflections with *I* > 2σ(*I*)
Graphite monochromator	*R*_int_ = 0.031
φ and ω scans	θ_max_ = 26.0°, θ_min_ = 2.3°
Absorption correction: multi-scan (*SADABS*; Sheldrick, 1996)	*h* = −26→25
*T*_min_ = 0.732, *T*_max_ = 0.797	*k* = −8→7
4975 measured reflections	*l* = −17→20

### Refinement

**Table:**

Refinement on *F*^2^	Primary atom site location: structure-invariant direct methods
Least-squares matrix: full	Secondary atom site location: difference Fourier map
*R*[*F*^2^ > 2σ(*F*^2^)] = 0.038	Hydrogen site location: inferred from neighbouring sites
*wR*(*F*^2^) = 0.101	H-atom parameters constrained
*S* = 1.01	*w* = 1/[σ^2^(*F*_o_^2^) + (0.0474*P*)^2^ + 1.3591*P*] where *P* = (*F*_o_^2^ + 2*F*_c_^2^)/3
1855 reflections	(Δ/σ)_max_ < 0.001
134 parameters	Δρ_max_ = 0.29 e Å^−^^3^
0 restraints	Δρ_min_ = −0.26 e Å^−^^3^

### Special details

**Table:**

Geometry. All e.s.d.'s (except the e.s.d. in the dihedral angle between two l.s. planes) are estimated using the full covariance matrix. The cell e.s.d.'s are taken into account individually in the estimation of e.s.d.'s in distances, angles and torsion angles; correlations between e.s.d.'s in cell parameters are only used when they are defined by crystal symmetry. An approximate (isotropic) treatment of cell e.s.d.'s is used for estimating e.s.d.'s involving l.s. planes.
Refinement. Refinement of *F*^2^ against ALL reflections. The weighted *R*-factor *wR* and goodness of fit *S* are based on *F*^2^, conventional *R*-factors *R* are based on *F*, with *F* set to zero for negative *F*^2^. The threshold expression of *F*^2^ > σ(*F*^2^) is used only for calculating *R*-factors(gt) *etc*. and is not relevant to the choice of reflections for refinement. *R*-factors based on *F*^2^ are statistically about twice as large as those based on *F*, and *R*- factors based on ALL data will be even larger.

### Fractional atomic coordinates and isotropic or equivalent isotropic displacement parameters (Å^2^)

**Table:**

	*x*	*y*	*z*	*U*_iso_*/*U*_eq_	Occ. (<1)
Cu1	0.2500	0.2500	0.0000	0.0438 (2)	
N1	0.22545 (12)	0.4838 (3)	0.04227 (16)	0.0377 (5)	
N2	0.16621 (13)	0.7649 (3)	0.03970 (17)	0.0406 (6)	
H2A	0.1312	0.8548	0.0199	0.049*	
C1	0.27220 (15)	0.5977 (4)	0.12627 (19)	0.0359 (6)	
C7	0.16303 (15)	0.5906 (4)	−0.00686 (19)	0.0369 (6)	
C6	0.23477 (16)	0.7750 (4)	0.1243 (2)	0.0386 (6)	
C2	0.34360 (16)	0.5579 (5)	0.2038 (2)	0.0480 (7)	
H2	0.3692	0.4396	0.2059	0.058*	
C9	0.03220 (15)	0.4427 (4)	−0.0953 (2)	0.0441 (7)	
H9A	0.0238	0.5342	−0.0548	0.053*	
H9B	−0.0124	0.4456	−0.1598	0.053*	
C10	0.04164 (16)	0.2279 (4)	−0.0559 (2)	0.0418 (7)	
C5	0.26760 (17)	0.9213 (5)	0.1980 (2)	0.0496 (8)	
H5	0.2430	1.0421	0.1954	0.059*	
C8	0.09664 (15)	0.5287 (5)	−0.1016 (2)	0.0439 (7)	
H8A	0.1118	0.4256	−0.1310	0.053*	
H8B	0.0789	0.6475	−0.1443	0.053*	
C3	0.37526 (18)	0.7010 (5)	0.2779 (2)	0.0520 (8)	
H3	0.4228	0.6775	0.3314	0.062*	
C4	0.33771 (18)	0.8787 (5)	0.2743 (2)	0.0532 (8)	
H4	0.3609	0.9718	0.3254	0.064*	
O2	−0.00441 (13)	0.1776 (3)	−0.03148 (19)	0.0594 (6)	
H2B	0.0009	0.0554	−0.0166	0.089*	0.50
O1	0.08916 (14)	0.1136 (4)	−0.05134 (19)	0.0717 (7)	

### Atomic displacement parameters (Å^2^)

**Table:**

	*U*^11^	*U*^22^	*U*^33^	*U*^12^	*U*^13^	*U*^23^
Cu1	0.0501 (3)	0.0312 (3)	0.0496 (3)	0.0076 (2)	0.0261 (3)	−0.0045 (2)
N1	0.0411 (12)	0.0305 (13)	0.0430 (13)	0.0056 (10)	0.0234 (11)	0.0001 (10)
N2	0.0450 (13)	0.0300 (13)	0.0523 (15)	0.0121 (11)	0.0295 (12)	0.0029 (11)
C1	0.0414 (15)	0.0297 (15)	0.0442 (16)	0.0002 (12)	0.0278 (13)	−0.0006 (12)
C7	0.0421 (15)	0.0327 (15)	0.0406 (15)	0.0062 (13)	0.0250 (13)	0.0068 (12)
C6	0.0456 (16)	0.0323 (16)	0.0483 (16)	0.0016 (13)	0.0319 (14)	0.0006 (13)
C2	0.0433 (16)	0.0448 (19)	0.0552 (19)	0.0062 (14)	0.0256 (15)	0.0004 (15)
C9	0.0420 (16)	0.0406 (18)	0.0459 (17)	0.0105 (13)	0.0207 (14)	0.0035 (13)
C10	0.0392 (15)	0.0414 (18)	0.0429 (16)	0.0052 (14)	0.0203 (13)	−0.0018 (13)
C5	0.061 (2)	0.0399 (18)	0.061 (2)	−0.0009 (15)	0.0415 (18)	−0.0099 (15)
C8	0.0484 (16)	0.0423 (18)	0.0426 (16)	0.0092 (14)	0.0252 (14)	0.0075 (13)
C3	0.0431 (17)	0.062 (2)	0.0504 (18)	−0.0074 (15)	0.0242 (15)	−0.0065 (16)
C4	0.058 (2)	0.055 (2)	0.0547 (19)	−0.0152 (17)	0.0351 (17)	−0.0184 (16)
O2	0.0629 (14)	0.0438 (13)	0.0855 (17)	−0.0025 (12)	0.0489 (14)	−0.0009 (13)
O1	0.0807 (16)	0.0472 (15)	0.111 (2)	0.0285 (13)	0.0671 (16)	0.0266 (14)

### Geometric parameters (Å, º)

**Table:**

Cu1—N1^i^	1.851 (2)	C9—C8	1.526 (4)
Cu1—N1	1.851 (2)	C9—H9A	0.9700
N1—C7	1.321 (3)	C9—H9B	0.9700
N1—C1	1.399 (3)	C10—O1	1.220 (3)
N2—C7	1.343 (3)	C10—O2	1.273 (3)
N2—C6	1.373 (4)	C5—C4	1.366 (4)
N2—H2A	0.8600	C5—H5	0.9300
C1—C2	1.385 (4)	C8—H8A	0.9700
C1—C6	1.388 (4)	C8—H8B	0.9700
C7—C8	1.488 (4)	C3—C4	1.384 (4)
C6—C5	1.392 (4)	C3—H3	0.9300
C2—C3	1.381 (4)	C4—H4	0.9300
C2—H2	0.9300	O2—H2B	0.8200
C9—C10	1.504 (4)		
			
N1^i^—Cu1—N1	180.00 (13)	C10—C9—H9B	108.2
C7—N1—C1	106.0 (2)	C8—C9—H9B	108.2
C7—N1—Cu1	126.43 (19)	H9A—C9—H9B	107.3
C1—N1—Cu1	127.13 (17)	O1—C10—O2	124.5 (3)
C7—N2—C6	108.4 (2)	O1—C10—C9	120.7 (3)
C7—N2—H2A	125.8	O2—C10—C9	114.8 (2)
C6—N2—H2A	125.8	C4—C5—C6	116.7 (3)
C2—C1—C6	120.6 (3)	C4—C5—H5	121.7
C2—C1—N1	130.9 (3)	C6—C5—H5	121.7
C6—C1—N1	108.5 (2)	C7—C8—C9	114.6 (2)
N1—C7—N2	111.5 (2)	C7—C8—H8A	108.6
N1—C7—C8	125.2 (2)	C9—C8—H8A	108.6
N2—C7—C8	123.3 (2)	C7—C8—H8B	108.6
N2—C6—C1	105.7 (2)	C9—C8—H8B	108.6
N2—C6—C5	132.5 (3)	H8A—C8—H8B	107.6
C1—C6—C5	121.9 (3)	C2—C3—C4	121.4 (3)
C3—C2—C1	117.3 (3)	C2—C3—H3	119.3
C3—C2—H2	121.3	C4—C3—H3	119.3
C1—C2—H2	121.3	C5—C4—C3	122.0 (3)
C10—C9—C8	116.5 (2)	C5—C4—H4	119.0
C10—C9—H9A	108.2	C3—C4—H4	119.0
C8—C9—H9A	108.2	C10—O2—H2B	109.5
			
C7—N1—C1—C2	179.1 (3)	C2—C1—C6—C5	1.7 (4)
Cu1—N1—C1—C2	−8.4 (4)	N1—C1—C6—C5	−179.1 (2)
C7—N1—C1—C6	0.0 (3)	C6—C1—C2—C3	0.0 (4)
Cu1—N1—C1—C6	172.48 (18)	N1—C1—C2—C3	−179.0 (3)
C1—N1—C7—N2	−0.2 (3)	C8—C9—C10—O1	−16.7 (4)
Cu1—N1—C7—N2	−172.78 (17)	C8—C9—C10—O2	165.2 (3)
C1—N1—C7—C8	−179.9 (2)	N2—C6—C5—C4	178.6 (3)
Cu1—N1—C7—C8	7.5 (4)	C1—C6—C5—C4	−2.2 (4)
C6—N2—C7—N1	0.4 (3)	N1—C7—C8—C9	103.1 (3)
C6—N2—C7—C8	−180.0 (2)	N2—C7—C8—C9	−76.5 (3)
C7—N2—C6—C1	−0.4 (3)	C10—C9—C8—C7	−74.7 (3)
C7—N2—C6—C5	178.9 (3)	C1—C2—C3—C4	−1.0 (4)
C2—C1—C6—N2	−179.0 (2)	C6—C5—C4—C3	1.2 (4)
N1—C1—C6—N2	0.2 (3)	C2—C3—C4—C5	0.4 (5)

Symmetry code: (i) −*x*+1/2, −*y*+1/2, −*z*.

### Hydrogen-bond geometry (Å, º)

**Table:**

*D*—H···*A*	*D*—H	H···*A*	*D*···*A*	*D*—H···*A*
N2—H2*A*···O1^ii^	0.86	1.97	2.725 (3)	146
O2—H2*B*···O2^iii^	0.82	1.69	2.491 (5)	166

Symmetry codes: (ii) *x*, *y*+1, *z*; (iii) −*x*, −*y*, −*z*.
